# PI3K Promotes Basal Cell Carcinoma Growth Through Kinase-Induced p21 Degradation

**DOI:** 10.3389/fonc.2021.668247

**Published:** 2021-06-29

**Authors:** Rachel Y. Chow, Ung Seop Jeon, Taylor M. Levee, Gurleen Kaur, Daniel P. Cedeno, Linda T. Doan, Scott X. Atwood

**Affiliations:** ^1^ Department of Developmental and Cell Biology, University of California, Irvine, Irvine, CA, United States; ^2^ Department of Dermatology, University of California, Irvine, Irvine, CA, United States; ^3^ Chao Family Comprehensive Cancer Center, University of California, Irvine, Irvine, CA, United States

**Keywords:** basal cell carcinoma, hedgehog, PI3K - AKT pathway, p21, atypical PKCι

## Abstract

Basal cell carcinoma (BCC) is a locally invasive epithelial cancer that is primarily driven by the Hedgehog (HH) pathway. Advanced BCCs are a critical subset of BCCs that frequently acquire resistance to Smoothened (SMO) inhibitors and identifying pathways that bypass SMO could provide alternative treatments for patients with advanced or metastatic BCC. Here, we use a combination of RNA-sequencing analysis of advanced human BCC tumor-normal pairs and immunostaining of human and mouse BCC samples to identify a PI3K pathway expression signature in BCC. Pharmacological inhibition of PI3K activity in BCC cells significantly reduces cell proliferation and HH signaling. However, treatment of *Ptch1^fl/fl^*; *Gli1*-*Cre^ERT2^* mouse BCCs with the PI3K inhibitor BKM120 results in a reduction of tumor cell growth with no significant effect on HH signaling. Downstream PI3K components aPKC and Akt1 showed a reduction in active protein, whereas their substrate, cyclin-dependent kinase inhibitor p21, showed a concomitant increase in protein stability. Our results suggest that PI3K promotes BCC tumor growth by kinase-induced p21 degradation without altering HH signaling.

## Introduction

The Hedgehog (HH) pathway is an evolutionarily conserved signaling pathway that plays an essential role in vertebrate embryogenesis and adult tissue homeostasis (1). Aberrant activation of the HH pathway results in uncontrolled proliferation and differentiation that leads to tumorigenesis in various tissues, with medulloblastoma (2, 3), rhabdomyosarcoma (4), and basal cell carcinoma (BCC) (5) commonly displaying mutations in HH pathway components. BCC is a locally invasive epithelial cancer that represents the most prevalent cancer in the United States, with more than four million cases estimated each year (6). While most cases of BCC are characterized by low mortality and metastasis that can be easily excised (7), advanced BCCs display elevated invasiveness, metastasis, and mortality (8). As the initiation and progression of BCCs predominantly depend on deregulation of the canonical HH pathway *via* activation of the seven-pass transmembrane protein Smoothened (SMO) (9), such dependency has led to the development of vismodegib and other SMO inhibitors for the treatment of locally advanced and metastatic BCCs (10). Yet, despite vismodegib demonstrating feasibility and efficacy in clinical trials against HH-driven medulloblastoma (11, 12), it has failed to achieve adequate success in treating advanced BCCs with 57% of patients displaying inherent resistance to the treatment (13) and 21% of treated patients developing secondary resistance after 56 weeks (14). Thus, it is vital to elucidate the mechanisms by which resistant BCCs evade SMO inhibition, as well as develop alternative therapeutic strategies that would effectively undermine such mechanisms.

In the normal vertebrate cell state, ion-driven cholesterol transporter Patched1 (PTCH1) actively depletes cholesterol from the membrane of the primary cilium and thus inhibits the cholesterol-dependent activation of SMO (15). This inhibition allows Suppressor of Fused homolog (SUFU) to sequester the Glioma-associated oncogene (GLI) transcription factors (16) and facilitate their post-translational proteolytic processing into repressor forms (17). The canonical HH pathway signaling initiates with the binding of HH ligands to PTCH1, which inhibits its activity and allows the activation of SMO *via* cholesterylation (18, 19). Activated SMO in turn induces the disassociation of the SUFU-GLI complex and facilitates the nuclear localization of the activator forms of GLI (20), which results in the expression of HH target genes. Uncontrolled activation of the HH pathway in BCC patients has been observed to occur primarily through inactivating mutations in PTCH1 (73%) or activating mutations in SMO (20%) (21). Yet, inspection of BCC patients with inherent and secondary resistance to vismodegib has revealed that the majority of mutations are within the SMO gene and either incite constitutive activity or deter inhibitor binding (22). Current efforts to circumvent BCC chemoresistance are focused on perturbing the oncogenic activity of GLI, either through directly inhibiting the GLI proteins or inhibiting the molecules that modulate GLI activity (23). Recent studies have demonstrated the potency of inhibiting GLI (24), DYRK1B (25), HDAC1 (26), BRD4 (27), MLK1 (28), and aPKC (29) in attenuating resistant BCCs in preclinical studies.

Recently, various studies have highlighted the critical interconnections between the HH pathway and other signaling pathways in promoting the persistence and chemoresistance of cancer. In BCC, progression and therapeutic resistance have been linked with molecular crosstalk between the HH pathway and other developmental signaling pathways such as WNT (30), Notch (31), TGF-β (32), and RAS/MAPK (33) pathways. The phosphoinositide 3-kinase (PI3K) pathway is another developmental signaling pathway that has been demonstrated to interact with the HH pathway in colon (34), pancreatic, and ovarian carcinomas (35). In addition, the PI3K pathway functions in therapeutic resistance against SMO inhibitors in medulloblastoma (36) and esophageal adenocarcinoma (37). Combinatory inhibition of both PI3K and HH signaling pathways in preclinical studies on medulloblastoma have demonstrated favorable efficacy in attenuating SMO inhibitor-resistant tumors (36), although the effects on other HH-mediated cancers like BCC remains to be determined.

The canonical PI3K pathway initiates with the activation of PI3K by receptor tyrosine kinases, which subsequently activates AKT *via* phosphorylation facilitated by phosphoinositides (38). Activated AKT in turn phosphorylates and regulates the activities of a wide array of signaling proteins that are associated with proliferation and differentiation of the cell (39). The PI3K/AKT pathway has been shown to interact with the HH pathway through multiple mechanisms that are largely independent of canonical HH signaling, with components of the PI3K/AKT pathway coinciding primarily upstream of GLI (40). In embryonic fibroblasts, upregulation of the PI3K/AKT pathway signaling promotes HH signaling by antagonizing the inhibitory function of PKA on GLI2 (41). Additionally, upregulation of the PI3K/AKT pathway signaling has been shown to promote HH signaling and tumor cell proliferation in esophageal (42) and breast cancers (43). However, the PI3K/AKT pathway has been shown to promote tumor cell growth with no effect on GLI1 activity in neuroblastomas (44), suggesting that PI3K/AKT either operates in parallel to or downstream of the HH pathway in this context. Thus, variation in how PI3K/AKT operates with respect to HH signaling confounds our ability to generally apply its function across distinct cancers.

Here, we demonstrate that the PI3K pathway signaling is upregulated in bulk-level RNA-sequencing data of 14 matched tumor-normal pairs. Human and mouse BCC tumors show a significant increase in PI3K protein expression, and PI3K is essential for both BCC tumor cell growth and HH signaling. However, our data shows disparate results between BCC cells and *in vivo* tumors, where PI3K inhibition has no effect on GLI1 activity despite suppressing tumor growth. Finally, we show that PI3K likely functions in BCC tumors by promoting aPKC- and AKT1-depedent degradation of cyclin-dependent kinase inhibitor p21 to maintain cell cycle progression. Our results suggest that the PI3K pathway functions in parallel to or downstream of the HH pathway to promote BCC tumor growth.

## Materials and Methods

### Ethics Statement

Human clinical studies were approved by the Ethics Committee of the University of California, Irvine. All human studies were performed in strict adherence to the Institutional Review Board (IRB) guidelines of the University of California, Irvine (2009–7083).

### Data Availability Statement

The data that supports the findings of this study are available in GEO at http://www.ncbi.nlm.nih.gov/geo/query/acc.cgi?acc-GSE58375, reference number GSE58375.

### RNA-Sequencing Analysis

RNA-sequencing data were obtained from patient-matched advanced human BCC patients (22). RNA-sequencing data were aligned as previously described (22). The NCBI Reference Sequence databases were used as reference annotations to calculate the values of reads per kilobase of transcript per million mapped reads for known transcripts (RPKM). RPKM values were then log2-transformed, and heat map analysis was used to visualize the differential gene expression. Pathway enrichment terms from the RNA sequencing data were obtained using Enrichr (45).

### Human Samples

Written informed consent was obtained for all archived human samples and was reviewed by the University of California, Irvine IRB. Human normal epidermis and BCC samples were collected from the UC Irvine Medical Center. Paraffinized samples were sectioned with a rotary microtome (Leica RM2155) at 7 μm for analysis. Samples were deparaffinized as described by Abcam, and antigen retrieval was performed using Tris-EDTA buffer (10 nM Tris base, 1 mM EDTA, 0.05% Tween-20, pH 9.0) at 60°C overnight.

### Cell Culture

ASZ001 cells (46) were grown in 154CF medium (Life Technologies) containing 2% Fetal Bovine Serum (FBS; Life Technologies) chelated overnight with Chelex® 100 Resin (Bio-Rad), 1% Penicillin-Streptomycin (P/S; Life Technologies), and 0.07 mM CaCl_2_. NIH3T3 cells (ATCC, CRL-1658) were grown in DMEM medium (Life Technologies) containing 10% FBS and 1% Penicillin-Streptomycin.

### RT-qPCR

ASZ001 cells at confluence were serum-starved with dimethyl sulfoxide (DMSO; Fisher Scientific) or varying concentrations of LY294002 (1 μM, 5 μM, 25 μM, and 100 μM; Fisher Scientific) or BKM120 (250 nM, 1.25 μM, 6.25 μM, and 31.25 μM; Fisher Scientific) for 24 hours. RNA was purified using Direct-zol RNA MiniPrep Plus (Zymo Research). Quantitative real-time polymerase chain reaction (RT-qPCR) was performed using the iTaq Universal SYBR Green 1-Step Kit (Bio-Rad) on the StepOnePlus Real-time PCR System (Applied BioSystem) using primers for *Gli1* (forward: 5’-GCAGGTG TGAGGCC AGGTAG TGACGA TG-3’, reverse: 5’-CGCGGG CAGCAC TGAGGA CTTGTC-3’) and *Gapdh* (forward: 5’-AATGAA TACGGC TACAGC AACAGG GTG-3’, reverse: 5’-AATTGT GAGGGA GATGCT CAGTGT TGGG-3’). Fold change in *Gli1* mRNA expression was measured using ΔΔCt analysis with *Gapdh* as an internal control. Experiments were run in triplicates and were repeated three times.

### MTT Assay

ASZ001 cells were seeded at 2000 cells/well into 96-well plates. After 48 hours, cells were treated with DMSO or varying concentrations of LY294002 (1 μM, 5 μM, 25 μM, and 100 μM) or BKM120 (250 nM, 1.25 μM, 6.25 μM, and 31.25 μM) for 2, 4, and 6 days. Growth assays were performed with MTT (Sigma-Aldrich) per manufacturer’s protocol. Plates were analyzed using the BioTek uQuant MQX200 Microplate Reader (BioTek). Experiments were run in 6 wells and were repeated three times.

### Mice

All mice were housed under standard conditions, and animal care was in compliance with the protocols approved by the Institutional Animal Care and Use Committee (IACUC) at the University of California, Irvine. *Ptch1^fl/fl^; Gli1-Cre^ERT2^* mice were administered with 100 μL of 10 mg/mL tamoxifen (Sigma) intraperitoneally for three consecutive days at six weeks of age. After five weeks when BCC microtumors have developed, mice were treated with 100 μL of DMSO or BKM120 (10 mg/kg) intraperitoneally for seven consecutive days. At the end of treatment, mice were euthanized and collected for their back skin. Collected skin samples were fixed with 4% paraformaldehyde (PFA; Electron Microscopy Sciences) for 30 minutes at room temperature, washed with DPBS (Life Technologies), immersed in 30% sucrose at 4°C overnight, and frozen in Tissue-Tek OCT Compound (Sakura Finetek). Samples were then cryo-sectioned using the CryoStar NX50 Cryostat (Thermo Fisher Scientific) at 14 μm for analysis. Five mice were used for each treatment condition.

### Microtumor Assessment

Skin sections were stained with hematoxylin and eosin (H&E; Richard-Allan Scientific) per standardized protocol. Stained sections were imaged at 200x magnification using the AmScope microscope with the AmScope MU500B digital camera. Tumor sizes were measured using ImageJ. BCC tumors display characteristic features such as peripheral basal palisading and are connected to the upper and lower bulge of the hair follicle, but not the infundibulum or matrix cells of anagen hair follicles. Microtumors were assessed as the total tumor size per square area. More than 50 tumors were measured from each of the five mice. Palpable macrotumors do not form in this genetic background unless additional genetic insults occur.

### Immunofluorescence

Skin sections were blocked using 10% Bovine Serum Albumin (BSA; Fisher Scientific) and 0.1% Triton X-100 (Fisher Scientific) in DPBS for 1 hour at room temperature. Sections were immunostained per standardized protocol using the following antibody dilutions: rabbit anti-PI3K (1:100, Abcam, ab40776), rabbit anti-GLI1 (1:500, Santa Cruz Biotechnology, sc-20687), rabbit anti-p-T304 GLI1 (1:200) (47), rabbit anti-AKT (1:400, Cell Signaling, 4691S), rabbit anti-p-T308 AKT (1:400, Cell Signaling, 13038S), rabbit anti-p21 (1:250, Cell Signaling, 2947S), and rabbit anti-p-T145 p21 (1:250, GeneTex, GTX32376). Sections were mounted in Prolong Diamond AntiFade Mountant with DAPI (Invitrogen). Immunostained sections were imaged using the Zeiss LSM700 confocal microscope (Zeiss) with 63x oil immersion objective. Pixel intensities were measured and averaged over five distinct tumors for each skin section using ImageJ. Images were arranged using ImageJ and Adobe Illustrator.

### Statistics

Statistical analyses were done with two-tailed t-test or one-way and two-way ANOVA using GraphPad Prism.

## Results

### PI3K/AKT Pathway Is Upregulated in Advanced BCC Tumors

**Figure 1:**
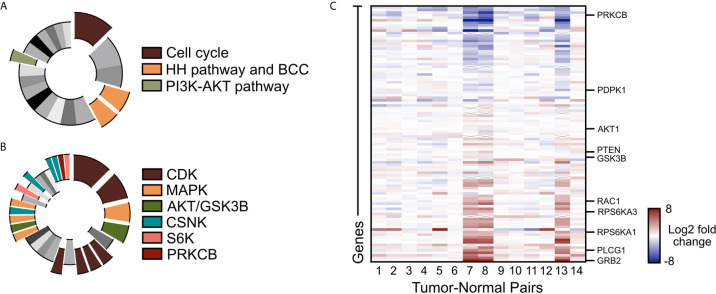
PI3K/AKT pathway is upregulated in advanced BCC tumors. **(A)** KEGG analysis of the upregulated genes in advance BCC tumors highlighting the significant indicated terms. Cell cycle, p = 3.10 x 10^-8^; BCC, p = 1.03 x 10^-4^; HH signaling pathway, p = 2.49 x 10^-4^; PI3K-AKT signaling pathway, p = 0.00675. **(B)** Kinase Enrichment Analysis of differentially expressed genes showing significant kinases as indicated. In descending significance to color codes: CDK2, p = 4.80 x 10^-12^; CDK1, p = 1.13 x 10^-8^; MAPK14, p = 2.59 x 10^-6^; GSK3B, p = 5.42 x 10^-6^; CDK15, p = 3.88 x 10^-4^; CDK14, p = 4.39 x 10^-4^; CDK18, p = 4.94 x 10^-4^; CDK11A, p = 6.23 x 10^-4^; MAPK1, p = 0.00460; AKT1, p = 0.00534; MAP3K10, p = 0.00641; CSNK2A1, p = 0.00796; MAPK9, p = 0.00828; RPS6KA5, p = 0.0123; CSNK2A2, p = 0.0165; CSNK1E, p = 0.0268; CSNK1D, p = 0.0275; PRKCB, p = 0.0281; RPS6KA1, p = 0.0332. **(C)** Heat map of the differentially expressed PI3K pathway genes in advanced human BCCs compared to patient-matched normal skin. X mark, absence of data.

To assess alternative pathways that may drive BCC tumor growth, we reanalyzed our bulk-level RNA-sequencing (RNA-seq) data of 14 matched tumor-normal pairs of advanced and SMO inhibitor-resistant BCC samples (22, 48). Differential gene expression analysis across the 14 tumor-normal pairs identified 1602 genes that were upregulated by two-fold or more in the resistant BCC tumors compared to their normal skin counterparts (48). Database analysis of the upregulated genes with the Kyoto Encyclopedia of Genes and Genomes (KEGG) showed the expected upregulation of the cell cycle, HH pathway, and BCC-associated genes (**Figure 1A**, **Supplementary Data 1**). Another term that was significantly enriched was the PI3K/AKT pathway (**Figure 1A** and **Supplementary Data 1**). Analogously, Kinase Enrichment Analysis (KEA) (49) linked many of the upregulated genes with kinases that are closely associated with PI3K/AKT signaling, such as MAPK, AKT, GSK3β, CSNK, S6K, and PRKCB (**Figure 1B** and **Supplementary Data 1**). Close analysis of the PI3K pathway gene expression showed many components and downstream targets significantly upregulated in most tumors, including GRB2, PLCG1, and RPS6KA1 (**Figure 1C** and **Supplementary Data 2**).

### PIK3CA Is Upregulated in Human and Mouse BCC Tumors

**Figure 2:**
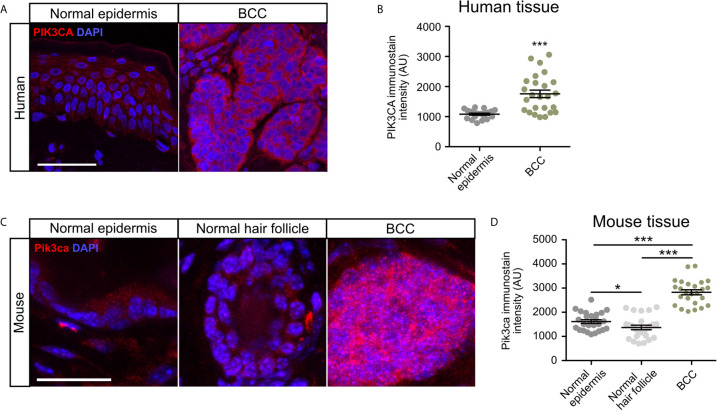
PIK3CA is upregulated in human and mouse BCC tumors. **(A)** Immunofluorescence staining of PI3KCA (red) and DAPI counterstain (blue) in human normal epidermis and nodular BCC tumors. Scale bar, 50 μm. **(B)** Quantification of PI3KCA immunofluorescence intensity (five points of measurement per sample, n=4 samples). AU, arbitrary unit. Error bar, SEM. **(C)** Immunofluorescence staining of Pi3kca (red) and DAPI counterstain (blue) in mouse normal epithelium, normal hair follicle, and BCC tumors. Scale bar, 25 μm. **(D)** Quantification of Pi3kca immunofluorescence intensity (five points of measurement per animal, n = 5 mice). AU, arbitrary unit. Error bar, SEM. Significance was determined by unpaired two-tailed *t* test. *p < 0.05; ***p < 0.001.

To validate whether PI3K pathway upregulation in BCC tumors is consistent at the protein level, we measured the expression of the catalytic subunit PIK3CA in human nodular BCC tumors and normal epidermis using immunofluorescence staining. We observed that tumors displayed significantly enhanced expression of PIK3CA compared to normal epidermis (**Figures 2A, B**). To analyze Pik3ca expression in mice, we utilized a *Ptch1^fl/fl^; Gli1-Cre^ERT2^* mouse model in which BCC microtumors arise from the hair follicle, secondary hair germ, and the touch dome in the interfollicular epidermis (50). BCC tumors were grown for five weeks post-Cre induction and formed predominantly from the hair follicle regions. Similar to our observations in human BCC tumors, we also observed a significant increase in Pik3ca expression in mouse BCC tumors compared to both normal epithelium and the hair follicle (**Figures 2C, D**). Together, these results suggest that PI3K pathway activity is upregulated in human and mouse BCC tumors.

### Inhibition of PI3K Suppresses Growth and Hh Signaling in BCC Cells *In Vitro*

**Figure 3:**
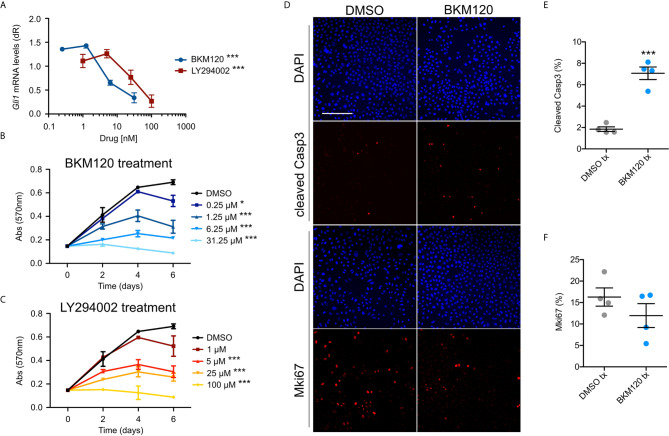
Inhibition of PI3K suppresses BCC cell growth and HH signaling. **(A)** *Gli1* mRNA expression in ASZ001 cells treated with DMSO or varying concentrations of BKM120 or LY294002 (n = 3 experiments). dR, delta reporter gene normalized to passive reference dye. Error bar, SEM. Significance was determined by one-way ANOVA test. ***P < 0.001. **(B, C)** MTT assay of ASZ001 cells treated with DMSO or varying concentrations of **(B)** BKM120 or **(C)** LY294002 (n = 3 experiments). Abs, absorbance. Error bar, SEM. Significance was determined by two-way ANOVA test. *p < 0.05; ***p < 0.001. **(D)** Immunofluorescence staining of the indicated markers in ASZ001 cells treated with DMSO or BKM120. Scale bar, 200 µm. **(E)** Quantification of cleaved Casp3 signal (n = 4 experiments). **(F)** Quantification of Mki67 signal (n = 4 experiments). Error bars, SEM. Significance was determined by unpaired two-tailed *t* test. ***p < 0.001.

To assess whether upregulation of the PI3K pathway signaling affects growth and HH signaling in BCCs, we assayed ASZ001 mouse BCC cells with two PI3K inhibitors, BKM120 and LY294002. BKM120 acts as an allosteric inhibitor of PI3K (51) while LY294002 acts as an ATP-competitive inhibitor (52). Treatment of ASZ001 cells with BKM120 and LY294002 both significantly decreased HH signaling as assayed by *Gli1* mRNA expression (**Figure 3A**). Additionally, treatment of ASZ001 cells with BKM120 and LY294002 both resulted in complementary and dose-dependent reduction of tumor cell growth over time (**Figures 3B, C**). A significant increase in Casp3-mediated apoptosis was also observed upon BKM120 inhibition, with the proliferation marker Mki67 trending downward (**Figures 3D–F**). Together, these results show that the PI3K pathway promotes BCC cell growth upstream of the HH pathway.

### Inhibition of PI3K Suppresses Growth but Not Hh Signaling in BCC Tumors *In Vivo*

**Figure 4:**
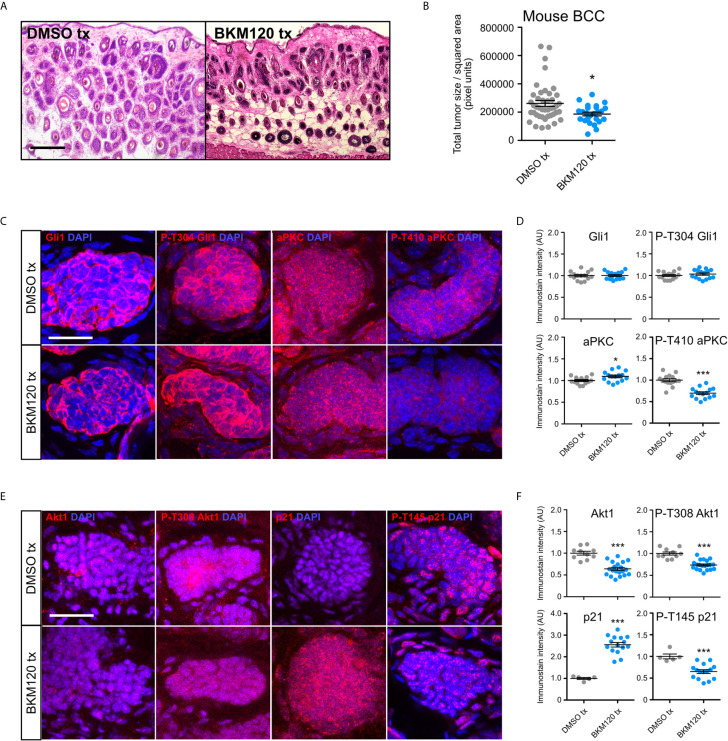
PI3K inhibition suppresses murine BCC growth and stabilizes p21. **(A)** Hematoxylin and eosin staining of dorsal back skin collected from *Ptch^fl/fl^; Gli1-Cre^ERT2^* mice treated with DMSO or BKM120. Tx, treatment. Scale bar, 50 μm. **(B)** Quantification of total tumor size per square area (n>250 tumors from 5 mice). Tx, treatment. **(C)** Immunofluorescence staining of indicated markers (red) and DAPI counterstain (blue) in *Ptch1^fl/fl^*; *Gli1*-*Cre^ERT2^* tumors treated with DMSO or BKM120. Scale bar, 25 μm. **(D)** Quantification of immunofluorescence intensity of indicated markers (five points of measurement per animal, n=3 mice). AU, arbitrary unit. **(E)** Immunofluorescence staining of indicated markers (red) and DAPI counterstain (blue) in *Ptch1^fl/fl^*; *Gli1*-*Cre^ERT2^* tumors treated with DMSO or BKM120. Scale bar, 25 μm. **(F)** Quantification of immunofluorescence intensity of indicated markers (five points of measurement per animal, n=3 mice). Error bars, SEM. Significance was determined by unpaired two-tailed *t* test. *p < 0.05; ***p < 0.001.

To evaluate whether inhibition of PI3K can serve as an effective therapeutic strategy in attenuating BCC tumors, we generated BCC tumors in the *Ptch1^fl/fl^; Gli1-Cre^ERT2^* mouse model and intraperitoneally injected either DMSO or 10 mg/kg of BKM120 daily for seven days. Histological staining of the dorsal skin of BKM120-treated mice showed a significant reduction in total tumor size compared to DMSO controls (**Figures 4A, B**). Interestingly, Gli1 protein expression was not altered in BKM120-treated mice (**Figures 4C, D**), suggesting that the PI3K operates downstream or in parallel to the HH pathway *in vivo*, a result that is similar to Mtor inhibition (48). The discrepancy between our *in vitro* and *in vivo* results may indicate that the tumor microenvironment alters how the PI3K pathway functions in relation to the HH pathway.

To further define how the PI3K pathway functions *in vivo*, we assayed that status of aPKC, a Gli1 kinase that is necessary for high sustained Gli1 activity (29). Atypical PKCs are activated downstream of PI3K by Pdk1-dependent phosphorylation at T410 in a variety of cell types (53, 54). While we observed a slight increase in total aPKC immunostaining in BKM120-treated mouse BCC tumors, phosphorylation at T410 was significantly reduced (**Figures 4C, D**), indicating suppressed kinase activity. Although aPKC phosphorylates and activates Gli1 at residue T304 (29), we observed no change in p-T304 Gli1 expression (**Figures 4C, D**), reinforcing the possibility of PI3K’s role outside of HH signaling and suggesting that aPKC likely exerts its effects on another substrate. Akt1 is also activated downstream of PI3K by Pdk1-dependent phosphorylation at T308 (55), and we found a significant reduction in both total and p-T308 Akt1 expression in BKM120-treated mouse BCC tumors (**Figures 4E, F**). As both aPKC and Akt1 facilitate the degradation of cyclin-dependent kinase inhibitor p21 by phosphorylating T145 (56–58), we assayed p21 protein expression and found a substantial increase in p21 stability and a corresponding decrease in p21 phosphorylation (**Figures 4E, F**). Altogether, our data suggests PI3K likely facilitates BCC tumor growth by promoting cell cycle progression through aPKC- and Akt1-mediated p21 degradation.

## Discussion

PI3K appears to operate at distinct levels within the HH pathway depending on context. For instance, upregulation of the PI3K/AKT pathway promotes HH signaling in embryonic fibroblasts (41), esophageal cancer (42), and breast cancer (43). Alternatively, the PI3K/AKT pathway promotes tumor cell growth independent of GLI1 activity in neuroblastomas (44), suggesting that PI3K/AKT either operates in parallel to or downstream of the HH pathway in this context. Our results show HH signaling is dependent on PI3K signaling in BCC cells grown in culture, but not in BCC tumors. This discrepancy between cell culture and three-dimensional (3D) growth conditions is a relatively common phenomenon and has been shown for the AKT-MTOR pathway, where inhibition of AKT resulted in elevated ERK signaling in cell culture but reduced signaling in 3D culture (59). Why this occurs in our system remains unclear, but one explanation could be that BCC tumors receive an abundance of signals from the surrounding niche that are absent in cell culture and can compensate for the loss of PI3K signaling to maintain HH pathway activation. Another possibility could be that higher *in vivo* dosage of drug could eventually suppress HH signaling, although the current dosage is able to inhibit tumor growth. Nevertheless, PI3K likely operates downstream or in parallel to the HH pathway in BCC, similar to our results for MTOR in *Ptch1^fl/fl^; Gli1-Cre^ERT2^* BCCs (48) and consistent with models where MTOR acts downstream of the HH pathway in *Ptch1^+/−^*/*SKH-1* BCCs (60).

p21 is a potent universal cyclin-dependent kinase inhibitor that is activated downstream of p53 upon DNA damage or other cellular stresses and promotes G1 cell cycle arrest, which can lead to senescence or apoptosis (61). When p53 is present, p21 and p53 can act together to help correct DNA damage and preserve genome stability. However, when p53 is disrupted, p21 can promote genomic instability and escape from senescence (62). p21 degradation is facilitated by aPKC- and AKT1-dependent phosphorylation at T145 (56–58). As aPKC and AKT1 are both overexpressed in BCC and are required for tumor growth (29, 63), our results suggest that BCCs activate both kinases downstream of PI3K to promote cell cycle progression and continued tumor growth. PI3K inhibition significantly suppresses aPKC and Akt1 activity, likely leading to enhanced p21 stability, suppression of proliferation, and enhanced apoptosis.

Targeting the PI3K pathway in the clinic may be a viable option for BCC patients. In addition to the present study showing BKM120 efficacy on *Ptch1^fl/fl^; Gli1-Cre^ERT2^* BCCs, inhibition of the PI3K/AKT/MTOR pathway has been shown to suppress irradiated *Ptch1^+/-^; Krt14^CreER2^; p53^fl/fl^* BCCs with XL765, but not XL147 or GDC-0941 (64). In addition, suppressing MTOR activity using everolimus can suppress *Ptch1^fl/fl^; Gli1-Cre^ERT2^* BCCs through an aPKC-dependent process (48) and has been used in the clinic for compassionate treatment of BCCs in elderly patients who refused surgery and did not respond to alternative treatments (65). Combination therapy may also be crucial to treat advanced BCC patients. For instance, PI3K/MTOR inhibition can delay therapeutic resistance against SMO inhibitors in mouse models of cancer, including BKM120 cotreatment in HH-driven medulloblastoma (36) or cotreatment with the MTOR inhibitor RAD-001 in esophageal adenocarcinoma (37). In addition, SMO inhibitor-resistant mouse medulloblastoma are still sensitive to PI3K inhibition (66) and combination therapy with the GLI inhibitor GANT61 and PI3K/MTOR inhibitor PI103 synergistically inhibited tumors in a HH-driven rhabdomyosarcoma mouse model (67). Altogether, PI3K pathway-targeted therapies, solely or in combination with HH pathway inhibitors, may broaden our repository for treating advanced and SMO inhibitor-resistant BCCs.

## Data Availability Statement

Publicly available datasets were analyzed in this study. This data can be found here: The data that supports the findings of this study are available in GEO at http://www.ncbi.nlm.nih.gov/geo/query/acc.cgi?acc-GSE58375, reference number GSE58375.

## Ethics Statement

The animal study was reviewed and approved by Institutional Animal Care and Use Committee (IACUC) at the University of California, Irvine.

## Author Contributions

SA and RC conceived the project. SA supervised research. RC performed experiments. UJ, TL, GK, and DC quantified and immunostained tumor data. LD collected and annotated human clinical samples SA, UJ, and RC wrote the manuscript. All authors contributed to the article and approved the submitted version.

## Funding

The work is funded by NIH grant R01CA237563 (SXA) and ACS Research Scholar Award RSG-19-089-01-DDC (SA).

## Disclaimer

The content is solely the responsibility of the authors and does not necessarily represent the official views of the NIH.

## Conflict of Interest

The authors declare that the research was conducted in the absence of any commercial or financial relationships that could be construed as a potential conflict of interest.
